# Epidemiological Findings of Alcohol Misuse and Dependence Symptoms among Adolescent Girls and Young Women Involved in High-Risk Sexual Behavior in Kampala, Uganda

**DOI:** 10.3390/ijerph17176129

**Published:** 2020-08-24

**Authors:** Yunia Mayanja, Onesmus Kamacooko, Daniel Bagiire, Gertrude Namale, Janet Seeley

**Affiliations:** 1 Medical Research Council/ Uganda Virus Research Institute and London School of Hygiene and Tropical Medicine (MRC/UVRI & LSHTM) Uganda Research Unit, P.O. Box 49, Plot 51-59 Nakiwogo Road, Entebbe 256, Uganda; onesmus.kamacooko@mrcuganda.org (O.K.); daniel.bagiire@mrcuganda.org (D.B.); gertrude.namale@mrcuganda.org (G.N.); janet.seeley@lshtm.ac.uk (J.S.); 2 Department of Global Health and Development, London School of Hygiene and Tropical Medicine (LSHTM), Keppel Street London, London WC1E 7HT, UK

**Keywords:** adolescents, female sex workers, alcohol misuse, sub-Saharan Africa

## Abstract

Alcohol-related harms may be increased among adolescent girls and young women (AGYW) involved in sex work, yet data on alcohol misuse among AGYW in sub-Saharan Africa are still scarce. We conducted a cross-sectional study among 15–24-year-old AGYW from January 2013 to December 2018 in Kampala, Uganda and used the Alcohol Use Disorder Identification Test (AUDIT) to study alcohol use patterns and dependence symptoms (dependence score ≥4). Of 1440 participants (median age 21 years), 83.1% had less than secondary education, 79.8% reported ≥10 paying sexual partners in the past month, 46.0% had ever experienced intimate partner violence (IPV), and 20.6% were living with HIV. Overall, 59.9% scored ≥8 and 29.4% scored ≥16 on the AUDIT. Of 277 (15.8%) with dependence symptoms, 69.1% were screened alcohol dependent. An AUDIT score ≥8 was associated with older age, illicit drug use, experiencing IPV, inconsistent condom use with paying partners, and HIV sero-negativity. All factors remained associated with a higher score ≥16 except HIV status. Similarly, illicit drug use, experiencing IPV and inconsistent condom use were associated with dependence symptoms and, in addition, a higher number of paying sexual partners. Alcohol misuse is high in this population, they urgently need harmful substance use reduction interventions.

## 1. Introduction

Globally, alcohol consumption has increased over the past two decades in almost all regions except the World Health Organization (WHO) identified European Region. In some parts of sub-Saharan Africa (SSA), alcohol consumption is 20% more than the regional average of 32.8 g of pure alcohol per day [1]. Alcohol misuse has consequences at both the individual and societal level including causing 5.3% of global deaths, social and economic consequences, infectious and non-transmissible diseases, and injuries [1]. In SSA, these poor outcomes may be exacerbated by unrecorded consumption of large amounts of alcohol produced illegally from local informal establishments which are not only cheaper but may contain higher amounts of ethanol and potential contaminants [2]. In East Africa, reported statistics on alcohol use among adults aged ≥15 years are: alcohol use disorders (AUD; 4.0–7.1%), alcohol dependence (1.4–2.5%), and alcohol attributable fraction of all deaths (4.0–7.3%) [1]. Regional data are not usually disaggregated for young people and yet SSA has the world’s largest proportion (33%) of young people [3]. Young people are defined as individuals 10–24 years; they include adolescents (10–19 years) and youth (15–24 years) [4].

Studies on alcohol consumption among young people are urgently needed as their number is expected to continue to rise through 2050, particularly in low- and middle-income countries (LMICs) [5]. As young people transition to adulthood, they may experimentally use different types of alcohol and other substances, which may have acute consequences related to accidents and injuries, unprotected sex, and violence [6]. Worldwide, 155 million (26.5% of total) adolescents aged 15–19 years are current alcohol drinkers, although this age group is less often current drinkers than the corresponding general population aged 15 years or more; by age 20–24 years the gap has closed and young people drink to the same extent and regularity as the general population [1]. Early onset of alcohol use among young people and the resulting problems have been associated with mental health disorders [5] and may affect long term health outcomes [7]. Alcohol consumption among school-going adolescents in the United States has been associated with externalizing behaviors such as sensation seeking, conduct problems, and affiliation with delinquent peers while African American ethnicity and internalizing behaviors, such as anxiety, predicted lower alcohol consumption [8]. In addition findings from a recent study among 15–24-year-old university students in south-east Asia indicate that the prevalence of alcohol consumption is 20.3% and is associated with being male, smoking, peer alcohol consumption, and truancy [9]. In SSA, surveys among 12–24-year-old students and employed youth in Tanzania (using the Alcohol Use Disorder Identification Test (AUDIT) and timeline-follow-back-calendar (TLFB) methods), and disadvantaged slum dwellers in Uganda (Cut-Annoyed-Guilty-Eye-Opener (CAGE) questionnaire) showed the prevalence of alcohol use disorders to be in the ranges of 12–47% and 11–50%, respectively [10,11,12]. Alcohol use in these studies was associated with being male, having disposable income, and engaging in higher risk sexual behavior [10,12]. A recent study among young fisher-folk (15–24 years), a recognized key population in Uganda, used a range of measures to estimate alcohol use including AUDIT, TLFB, and phosphatidyl ethanol (PEth) blood levels with reliable results across all three measures. AUDIT scores ≥8 and PEth values ≥20 ng/mL were 19.4% and 20.5%, respectively [11]. Older age, low education, smoking, and infection with herpes simplex virus-2 were associated with the observed outcomes. Although data on alcohol dependence among young people are still limited, the prevalence reported among 15–19-year-old students in Brazil is 16.4%; associated factors included male gender and higher socioeconomic status [13].

The consequences of alcohol consumption among young key populations may be more pronounced among female sex workers (FSWs) because of their unique situations such as work-related exposure to alcohol [14,15]. Their social contexts also expose them to exploitation, limited social support, and intimate partner violence (IPV) [16] leaving them vulnerable to engaging in alcohol misuse. Many sex workers only start drinking alcohol when they join the trade on the advice of more experienced peers; alcohol helps them to cope with the job and develop courage in dealing with clients, it putatively protects women against the cold, and helps them to cope with the associated social stigma [14,15]. Consumption is also promoted in alcohol selling establishments such as bars which often also serve as sex work venues [17], in addition clients encourage consumption as they buy drinks for the women [14]. We have previously reported that recruiting clients from bars and night clubs was associated with problem drinking in a cohort that included adult FSWs [18]. However, data on alcohol misuse and dependence among AGYW involved in high-risk sexual behavior, including sex work, are lacking.

The aim of this study was to: (i) estimate the prevalence of alcohol use disorders and dependence symptoms (dependence score ≥4) using AUDIT, (ii) determine factors associated with alcohol use disorders, and (iii) determine the factors associated with alcohol dependence symptoms.

## 2. Methods

### 2.1. Study Design and Setting

We performed a cross-sectional analysis of records of 15–24-year-old AGYW enrolled into a cohort of high-risk women from January 2013 to December 2018. The cohort included women 15–49 years and enrolled at the Good Health for Women Project (GHWP) clinic located in a peri-urban community in southern Kampala. Field workers worked with community peer leaders to mobilize women from commercial hotspots from where we enrolled them into the GHWP clinic irrespective of HIV status. The clinic provides a comprehensive package of HIV prevention and treatment services which includes: HIV counselling and testing (HCT); free services including treatment for common illnesses, contraception, syndromic management of sexually transmitted infections (STIs), screening and counselling for alcohol use disorders, and intimate partner violence (IPV); free male and female condoms; and treatment for their male regular partners and children below 5 years. In addition, those living with HIV received tuberculosis screening and treatment, prophylaxis for opportunistic infections, and anti-retroviral therapy (ART).

### 2.2. Study Participant Eligibility Criteria

We selected baseline records of all participants enrolled from January 2013 to December 2018 who were aged 15–24 years at the time of enrolment. Participants <18 years met criteria as being mature or emancipated minors as per guidelines of the Uganda National Council for Science and Technology (UNCST) [19].

### 2.3. Study Outcomes and Measures

The main study outcome was prevalence of alcohol misuse. We determined this using the Alcohol Use Disorder Identification Test/AUDIT, which was developed by WHO and validated across several countries for identifying alcohol use disorders. The test determines alcohol use over the past 12 months and comprises of 10 screening questions covering the domains of: alcohol consumption (questions 1–3), drinking behavior/dependence (questions 4–6), and alcohol-related problems (questions 7–10) [20]. Questions 1 to 8 were scored on a five-point scale from 0, 1, 2, 3, and 4, while questions 9 and 10 were scored on a three-point scale from 0, 2, and 4. Scores for all 10 questions were summed up and total scores categorized as follows: 1–7 (low-risk drinking/abstinence), 8–15 (moderate-risk/hazardous drinking), 16–19 (high-risk/harmful drinking), and ≥20 (high-risk/alcohol dependent) [21].

Alcohol misuse was measured as a binary variable (Yes/No) at two levels:

Participants who scored ≥8 were categorized as moderate to high risk drinkers.
Participants who scored ≥16 were categorized as high-risk drinkers.

The secondary outcome was prevalence of dependence symptoms measured as a binary variable (Yes/No). It was derived from the sum of scores of questions 4–6. Presence of dependence symptoms was “Yes” for a score ≥4.

### 2.4. Independent Variables

Sociodemographic variables: age, marital status, education level, main job, and number of biological children.

Behavioral variables: ever used illicit drugs, contraceptive use, experience of IPV, condom use with paying partners one month prior to enrolment, history of ever testing for HIV, and duration since the last HIV test.

Clinical variables: HIV status and STI symptoms (syndromic diagnosis).

### 2.5. Laboratory Methods

Laboratory staff performed HIV testing on serum using two or more rapid diagnostic tests for antibodies to HIV as per the national guidelines approved at that time. From January 2013 to January 2018, the algorithm used “Determine” as the screening test, “Statpak” as the confirmatory test, and “Unigold” as the tiebreaker. From February 2018 onwards, “SD Bioline (Standard Diagnostics, Inc.,)” replaced “Unigold” as the tiebreaker test.

### 2.6. Data Collection

Trained study staff collected data using an electronic Microsoft Access database. The AUDIT tool and questionnaires to collect other data were designed and programmed into the database which was pilot tested before data collection/entry began.

### 2.7. Statistical Analysis

The project data manager cleaned the data and exported it to Stata 15 (StataCorp, College Station, TX, USA) for analysis. Participants’ categorical demographic and behavioral characteristics were summarized by counts and percentages. Continuous characteristics were summarized by means and standard deviations (SD) or medians and inter quartile ranges.

- We determined the proportion with AUD as the number that had AUDIT score ≥8 divided by the total number enrolled, and expressed as a percentage.
- Of those with AUD, we also determined the proportion who were high-risk drinkers as the number that had AUDIT score ≥16 divided by the total number enrolled, and expressed as a percentage.
- The proportion with dependence symptoms were those who had a score of ≥4 divided by the total number enrolled, and expressed as a percentage.

The proportion who had AUD (AUDIT ≥8 and AUDIT ≥16) were further analyzed by the different demographic and behavioral characteristics using chi-square tests. Logistic regression models were fitted to find factors associated with high-risk drinking and a high dependence score. Factors which were statistically significant at the unadjusted level of analysis (log likelihood ratio test (LRT), *p* < 0.20) were included in the multivariable logistic regression model. We used complete case analysis to run our final model. Age was considered as an a priori confounder. Factors were retained in the final multivariate logistic regression model if their inclusion did not make the model significantly worse at *p* < 0.05. From the literature, education level and HIV status have shown associations with alcohol misuse; thus, they were included in the final models. We present results as adjusted odds ratios (aOR) with 95% confidence intervals (CI).

### 2.8. Missing Data (Sensitivity Analysis)

We conducted a sensitivity analysis to ascertain if missing data on selected covariates had an impact on the study results. Data values were imputed for participants who had missing data using the multivariate imputation by chained equations approach [22]. A sample of missing values was created, conditional on the distribution of the remaining covariates in the adjusted model. We assumed that the data were missing at random and carried out 10 rounds of multiple imputations; the final data for analysis after imputation were combined using Rubin’s rule [23]. We compared the results from the complete case analysis and those from the imputed models to assess for significant differences.

### 2.9. Ethics Statement

All subjects gave their informed consent for inclusion before they participated in the study. The study was conducted in accordance with the Declaration of Helsinki, and the protocol was approved by the Uganda National Council for Science and Technology (HS 364) and Uganda Virus Research Institute-Research Ethics Committee (GC/127/14/10/30).

## 3. Results

From January 2013 to December 2018, we enrolled 5721 women involved in high-risk sexual behavior, of whom 1898 (33.2%) were AGYW aged 15–24 years. Of these, 458 had missing data on one or more variables and were dropped from the analysis; we analyzed data for 1440 who had complete records. The 458 with missing data were younger and had lower education than the 1440 retained and these differences were significant (*p* < 0.05), however the sensitivity analysis showed that the results from the imputed model were similar to those we present in this paper and are included as a supplementary file (Tables S1 and S2).

### 3.1. Baseline Characteristics of AGYW Enrolled at the GHWP Clinic in Kampala, Uganda

The median age of the 1440 retained participants was 21 years (interquartile range (IQR), 19–23 years), 83.1% had less than secondary education and 44.0% were separated/widowed. Mean age at first pregnancy was 17 years (SD ± 2.8), 69.8% had at least one child, and 66.9% reported using reliable contraception (commonly Depo Medroxy progesterone acetate (DMPA)).

HIV prevalence was 20.6% with HIV sero-positivity higher among the older participants 20–24-year-olds compared to 15–19-year-olds (23.9% vs. 10.8%; *p* ≤ 0.001). Most participants (92%) had been tested for HIV, of whom 72.0% had their last HIV test within 6 months prior to enrolment. Additionally, 79.8% reported ≥10 paying sexual partners in the month prior to enrolment and reported consistent condom use with paying partners was 49.1%. IPV was reported by 46.1%, mainly with casual partners (40.4% of those reporting IPV), 505 (35.1%) reported ever using illicit drugs, of whom 419 (83.0%) were current users, commonly khat and marijuana. Table 1 shows baseline participant characteristics.

### 3.2. Alcohol Consumption Patterns and Dependence Scores of AGYW

Of 1440 participants, 1086 (75.4%) reported currently using alcohol. AUDIT screening results were: low-risk drinking (40.1%), moderate-risk/hazardous drinking (30.5%), high-risk/harmful drinking (13.3%), and high-risk/alcohol dependent (16.1%).

Overall, 277 participants had a dependence score of ≥4. Of these, 69.1% were identified as alcohol dependent, 22.5% were harmful drinkers, and 8.4% hazardous drinkers (Table 2).

### 3.3. Prevalence of Alcohol Misuse (AUDIT ≥8 and AUDIT ≥16) by Sociodemographic and Behavioral Characteristics

Overall, 863 (59.9%) of participants had AUDIT score ≥8. An AUDIT score ≥8 was higher among participants who were older (20–24 years; 62.2 vs. 53.1; *p* = 0.002), reported IPV (65.3% vs. 55.4%; *p* < 0.001), had higher number of paying sexual partners in the past month (61.5% vs. 53.6%; *p* = 0.014), reported inconsistent condom use with paying partners (64.4 vs. 55.3%; *p* < 0.001), and used illicit drugs (77.6% vs. 52.7%; *p* < 0.001).

The proportion identified as high-risk drinkers (AUDIT score ≥16) was 24.9% (*n* = 424). AUDIT score ≥16 was higher among participants who reported IPV (37.9% vs. 22.2%; *p* < 0.001), had higher number of paying sexual partners (31.3% vs. 22.0%; *p* = 0.002), reported inconsistent condom use with paying partners (34.7% vs. 24.1%; *p* < 0.001), and used illicit drugs (46.5% vs. 22.4%; *p* < 0.001; Table 3).

### 3.4. Factors Associated with Alcohol Misuse among AGYW

Using the adjusted analysis, having an AUDIT score ≥8 was associated with being older (20–24 years; aOR 1.61; 95% CI 1.24–2.08), illicit drug use (aOR 3.04; 95% CI 2.32–3.98), ever experiencing IPV (aOR 1.35; 95% CI 1.07–1.69), inconsistent condom use with paying partners (aOR 1.44; 95% CI 1.15–1.80), and being HIV negative (aOR 1.52; 95% CI 1.15–2.01).

Using the adjusted analysis having an AUDIT score ≥16 was associated with being older (20–24 years; aOR 1.43; 95% CI 1.06–1.92), illicit drug use (aOR 2.80; 95% CI 2.18–3.61), ever experiencing IPV (aOR 1.87; 95% CI 1.46–2.39), and inconsistent condom use with paying partners (aOR 1.60; 95% CI 1.25–2.04; Table 4).

### 3.5. Factors Associated with Dependence Symptoms among AGYW

Using the adjusted analysis dependence was associated with illicit drug use (aOR 2.30; 95% CI 1.70–3.11), ever experiencing IPV (aOR 1.53; 95% CI 1.13–2.06), inconsistent condom use with paying partners (aOR 1.95; 95% CI 1.43–2.64), and having ≥10 sexual partners (aOR 1.69; 95% CI 1.09–2.63). There was borderline significance between HIV negative status and dependence (aOR 1.43; 95% CI 0.98–2.09; Table 5).

## 4. Discussion

Among AGYW in the present study the proportion with AUDIT ≥8 was higher than that reported among young people in fishing communities, another key population in East Africa [11]. The burden of alcohol misuse among the participants was even greater, as half of those who misused alcohol were in the high-risk category (AUDIT ≥16) and, not all of those with dependence symptoms were alcohol dependent on the AUDIT score. In the context of AGYW who earn money from sex work, the high prevalence of alcohol misuse reported here appears to result from consumption driven by emotional and socioeconomic needs.

Older age (20–24 years) was associated with alcohol misuse and dependence symptoms. The present findings are similar to other studies done among young people in Uganda [11,12]. Global reports also show that harmful drinking, while not prevalent in those below 19 years, increases in the 20–24 year age group [1]. The present study outcomes were also highly associated with current illicit drug use, an association that has also been found among young fisher folk in Uganda [11]. The most important physical, biological, and psychological development between childhood and adulthood occurs before the age of 20 years. During this period, adolescents may adopt impulsive behaviors as they experience new social roles and interact more with peers. One form of impulsivity, sensation seeking, rises dramatically during adolescence and increases risks to healthy development [24]. Alcohol and illicit drug use are some of the risky behaviors initiated during adolescence [25]; given their addictive nature, consumption patterns increase in young adulthood if there are no risk reduction interventions. In addition, the legal minimum age for alcohol consumption in Uganda is 18 years [1], the propensity to consume alcohol may increase subsequent misuse among young adults ≥20 years who already consume alcohol. Thus, AGYW need to be targeted early for substance use reduction interventions to mitigate harmful consequences of continued misuse later in life.

The present results indicate that participants with alcohol misuse and dependence symptoms were more likely to report a higher number of paying sexual partners and inconsistent condom use. Studies have shown an association between alcohol misuse and high-risk sexual behavior among young people [10,12] and older key populations [18,26]. Excessive alcohol use impairs cognitive function and hence may reduce one’s ability to use or negotiate condom use [27]. Furthermore, a qualitative study among adolescents showed that the effects of alcohol consumption on risky behavior are most prevalent when there is impaired judgment and complete loss of control [28]. For those involved in sex work, the need for resolve when dealing with many clients leads to heavy drinking sessions as a way of coping [14]. In situations where both FSWs and their clients consume alcohol, the potential for risky sexual practices increases and societal calls for initiating alcohol reduction interventions at the structural level are needed so that consequences such as unwanted pregnancies and risk of infection with HIV and sexually transmitted infections is not heightened.

The findings of the present study also showed that IPV was significantly associated with the alcohol misuse and dependence symptom outcomes. High-risk women generally experience high levels of IPV [29]. Acts of IPV remain unreported due to the criminalization of sex work in many parts of SSA [30]; disclosure rates are therefore low [31] and this undermines their ability to interact with society including community rehabilitation facilities where they could seek help. However, during times of intoxication, women may also be perpetrators of violence. Reports among young people have shown them to be both perpetrators and victims of IPV [32] and violence against other community members [33]. Counselling and empowering women leads to a reduction in IPV, HIV-risk behaviors, and HIV incidence, possibly through reduction in forced sex [34,35]. Community involvement is also important as it increases the likelihood of community members intervening when they witness IPV [36].

Motivational interviews may have a role in reducing the prevalence of alcohol misuse among AGYW involved in sex work. Despite the challenges posed by excessive alcohol use, two systematic reviews of randomized clinical trials that assessed brief alcohol interventions found that multi-contact brief counselling sessions reduced self-reported at-risk/harmful and hazardous alcohol use in primary care [37,38]. Brief motivational interviews delivered in a few sessions enable individuals to get education on alcohol use and health-related harms, identify high-risk situations for heavy drinking, and develop a personal plan and strategies to facilitate an individual’s motivation to reduce alcohol drinking [38].

## 5. Limitations and Strengths

Data from 458 of enrolled participants were excluded from the analysis because they had missing data for one or more variables. The missing data however did not affect our ability to detect associations with the outcomes; we performed a sensitivity analysis whose results did not differ from what we report in this paper. The cross-sectional study design precludes us from demonstrating causality between independent variables and the study outcomes.

The sample size we analyzed was large enough to result in the narrow confidence intervals observed in the statistical tests. This indicates that the sample may be representative and supports the study’s external validity.

## 6. Conclusions and Recommendations

Alcohol misuse is highly prevalent among AGYW involved in sex work and the possibility of continued misuse is high given both the nature of their work and the addictive properties of alcohol. The observed dependence scores have not been widely reported. However, the findings of the present study indicate that dependence can be detected earlier among those misusing alcohol, and counselling or motivational interviewing can be initiated before individuals become alcohol dependent.

Young key populations need health service delivery models that integrate counselling and referrals for alcohol misuse and high dependence scores. An advantage of the AUDIT tool is that it is easy to administer and therefore can be used in primary health care settings with lower cadre health staff; screening for alcohol use should be included in the routine health care at facilities with high turnover of young women seeking health care. Opportunities for such screening can be created at service points delivering sexual and reproductive health services, HIV prevention, care and treatment and immunization services. In addition, regular community outreach services can be targeted to reach young people who may be out of school and also unable to access the formal health facilities for example those in hotpots and slums. Community outreach services for alcohol screening can be integrated within already existing public awareness campaigns for example HIV testing campaigns or social events such as sports events and supported by broadcast or social media for publicity. Models should however be considered in the context of young people’s lives, to address the multiple levels of influence impacting their lives and those of their peers, sexual partners, owners of entertainments facilities in commercial hotspots, and community leaders. Interventions to reduce alcohol misuse among AGYW should also target illicit drug use and IPV at the individual and community levels. Such interventions empower women leading to a reduction in IPV and HIV-risk behaviors, and increase the likelihood of community members intervening when they witness IPV.

## Figures and Tables

**Table 1:** Baseline characteristics of adolescent girls and young women (AGYW) enrolled at the Good Health for Women Project (GHWP) clinic in Kampala, Uganda from January 2013 to December 2018.

Variable	Categories	Frequency (*N* = 1440)	Percent (%)
Age (Years)			
	15–19	360	25.0
	20–24	1080	75.0
Education level			
	None	82	5.7
	Less than secondary	1115	77.4
	Secondary or higher	243	16.9
Marital status			
	Single (never married)	746	51.8
	Married	61	4.2
	Widowed/separated	633	44.0
Number of biological children			
	None	435	30.2
	One	586	40.7
	>1	419	29.1
Ever used illicit drugs			
	Yes	505	35.1
	No	935	64.9
Ever tested for HIV			
	Yes	1325	92.0
	No	115	8.0
Last HIV test ^a^			
	≤6 months	954	72.0
	>6 months	371	28.0
HIV status			
	Positive	297	20.6
	Negative	1143	79.4
Main job			
	Sex work	972	67.5
	Other job	468	32.5
Paying sexual partners in past one month			
	<10 partners	291	20.2
	≥10 partners	1149	79.8
Condom use with paying partners in past month			
	Consistent	707	49.1
	Inconsistent	733	50.9
Ever experienced intimate partner violence (IPV)			
	Yes	662	46.0
	No	778	54.0
Contraceptive use			
	Yes	964	66.9
	No	476	33.1

^a^ Numbers include only those who had ever been tested for HIV.

**Table 2:** Alcohol consumption patterns and dependence symptoms among AGYW.

AUDIT Score	Risk Level	Alcohol Consumption Pattern	Dependence Symptoms (Dependence Score ≥4)
		Frequency (*N* = 1440) *n* (%)	Frequency (*n* = 227) *n* (%)
0–7	Low-risk drinking/Abstinence	577 (40.1)	0 (0)
8–15	Moderate-risk/hazardous drinking	439 (30.5)	19 (8.4)
16–19	High-risk/harmful drinking	192 (13.3)	51 (22.5)
≥20	High-risk/Alcohol dependent	232 (16.1)	157 (69.1)

AUDIT is Alcohol Use Disorder Identification test.

**Table 3:** Prevalence of alcohol use disorders among AGYW by sociodemographic and behavioral characteristics.

		AUDIT Score ≥8		AUDIT Score ≥16	
Variable	Sub-Category	*n* (col %)	χ^2^ *p*-value	*n* (col %)	χ^2^ *p*-value
Overall		863 (59.9)		424 (29.4)	
Age (years)			0.002		0.062
	15–19	191 (53.1)		92 (25.6)	
	20–24	672 (62.2)		332 (30.7)	
Education level			0.928		0.103
	Less than secondary	718 (60.0)		363 (30.3)	
	Secondary or higher	145 (59.7)		61 (25.1)	
Marital Status			0.07		0.271
	Single never married	441 (59.1)		206 (27.6)	
	Married	29 (47.5)		18 (29.5)	
	Separated or widowed	393 (62.1)		200 (31.6)	
Biological children			0.46		0.992
	None	267 (61.4)		128 (29.4)	
	≥1	596 (59.3)		195 (29.5)	
Current illicit drug use			<0.001		<0.001
	No	538 (52.7)		229 (22.4)	
	Yes	325 (77.6)		222 (46.5)	
Ever experienced IPV			<0.001		<0.001
	No	431 (55.4)		173 (22.2)	
	Yes	432 (65.3)		251 (37.9)	
Number of paying sexual partners in past month			0.014		0.002
	<10	156 (53.6)		64 (22.0)	
	≥10	707 (61.5)		360 (31.3)	
Consistent condom use with paying sexual partners			<0.001		<0.001
	No	472 (64.4)		254 (34.7)	
	Yes	391 (55.3)		170 (24.1)	
Contraceptive use			0.092		0.277
	No	300 (63.0)		149 (31.3)	
	Yes	563 (58.4)		275 (28.5)	
HIV status			0.232		0.949
	Positive	169 (56.9)		87 (29.3)	
	Negative	694 (60.7)		337 (29.5)	

**Table 4:** Factors associated with Alcohol misuse among AGYW.

		AUDIT Score ≥8			AUDIT Score ≥16		
Variable	Sub-Category	Unadjusted OR (95% CI)	LRT *p*-value	Adjusted OR (95% CI)	Unadjusted OR (95% CI)	LRT *p*-value	Adjusted OR (95% CI)
Age (years)						0.062	
	15–17	1.00	0.002	1.00	1.00		1.00
	20–24	1.46 (1.15–1.85)		1.61 (1.24–2.08)	1.29 (0.99–1.69)		1.43 (1.06–1.92)
Education level			0.928			0.104	
	Less than secondary	1.00		1.00	1.00		1.00
	Secondary or higher	0.99 (0.75–1.31)		1.03 (0.77–1.39)	0.77 (0.56–1.06)		0.83 (0.59–1.17)
Marital Status			0.277			0.106	
	Single never married	1.00		-	1.00		1.00
	Married	0.63 (0.37–1.06)		-	1.09 (0.62–1.94)		1.14 (0.62–2.07)
	Separated or widowed	1.13 (0.91–1.41)		-	1.21 (0.96–1.53)		1.03 (0.80–1.33)
Biological children			0.461			0.992	
	None	1.00		-	1.00		-
	≥1	0.92 (0.73–1.15)		-	1.00 (0.78–1.28)		-
Current illicit drug use			<0.001			<0.001	
	No	1.00		1.00	1.00		1.00
	Yes	3.10 (2.39–4.03)		3.04 (2.32–3.98)	3.01 (2.36–3.83)		2.80 (2.18–3.61)
Ever experienced IPV			<0.001			<0.001	
	No	1.00		1.00	1.00		1.00
	Yes	1.51 (1.22–1.87)		1.35 (1.07–1.69)	2.14 (1.69–2.69)		1.87 (1.46–2.39)
Paying partners (past month)			0.014			0.002	
	<10	1.00		1.00	1.00		1.00
	≥10	1.38 (1.07–1.79)		1.12 (0.85–1.48)	1.62 (1.19–2.19)		1.20 (0.87–1.66)
Condom use with paying sexual partners			<0.001			<0.001	
	Consistent	1.00		1.00	1.00		1.00
	Inconsistent	1.46 (1.18–1.81)		1.44 (1.15–1.80)	1.68 (1.33–2.11)		1.60 (1.25–2.04)
Contraceptive use			0.092			0.277	
	No	1.00		1.00	1.00		-
	Yes	0.82 (0.66–1.03)		0.83 (0.65–1.05)	0.88 (0.69–1.11)		-
HIV status			0.232			0.949	
	Positive	1.00		1.00	1.00		1.00
	Negative	1.17 (0.90–1.52)		1.52 (1.15–2.01)	1.01 (0.76–1.34)		1.27 (0.94–1.72)

OR= Odds ratio; CI= Confidence interval; LRT=Log likelihood ration test

**Table 5:** Factors associated with dependence symptoms among AGYW.

Variable	Sub-Category	Dependence Symptoms (Score ≥4)	Unadjusted OR	*p*-value	Adjusted OR
		*n* (col %)			
Overall		227 (15.8)			
Age (years)				0.337	
	15–19	51 (14.2)	1.00		1.00
	20–24	176 (16.3)	1.18 (0.84–1.65)		1.44 (0.99–2.07)
Education level				0.224	
	Less than secondary	195 (16.3)	1.00		1.00
	Secondary or higher	32 (13.2)	0.78 (0.52–1.17)		0.80 (0.52–1.22)
Marital status				0.514	
	Single never married	113 (15.2)	1.00		-
	Married	10 (16.4)	1.09 (0.54–2.23)		-
	Widowed/separated	104 (16.4)	1.10 (0.82–1.47)		-
Biological children				0.185	
	None	77 (17.7)	1.00		1.00
	≥1	150 (14.9)	0.82 (0.60–1.10)		0.75 (0.54–1.04)
Current illicit drug use				<0.001	
	No	110 (11.8)	1.00		1.00
	Yes	117 (23.2)	2.26 (1.69–3.01)		2.30 (1.70–3.11)
Ever experienced IPV				<0.001	
	No	94 (12.1)	1.00		1.00
	Yes	133 (20.1)	1.83 (1.37–2.44)		1.53 (1.13–2.06)
Paying sexual partners in past month				<0.001	
	<10 partners	27 (9.3)	1.00		1.00
	≥10 partners	200 (17.4)	2.06 (1.35–3.15)		1.69 (1.09–2.63)
Condom use with paying partners				<0.001	
	Consistent	148 (20.2)	1.00		1.00
	Inconsistent	79 (11.2)	2.01 (1.49–2.70)		1.95 (1.43–2.64)
Contraceptive use				0.217	
	No	67 (14.1)	1.00		-
	Yes	160 (16.6)	1.21 (0.89–1.65)		-
HIV status				0.495	
	Positive	43 (14.5)	1.00		1.00
	Negative	184 (16.1)	1.13 (0.79–1.62)		1.43 (0.98–2.09)

## Data Availability

The datasets used and analyzed during the current study are available from the corresponding author on reasonable request.

## Supplementary Materials

The following are available online at https://www.mdpi.com/1660-4601/17/17/6129/s1. Table S1, Table S2 showing results of the sensitivity analysis have been included in the submission as supplementary files.
