# Supplementary material for: Epidemiological Findings of Alcohol Misuse and Dependence Symptoms among Adolescent Girls and Young Women Involved in High-Risk Sexual Behavior in Kampala, Uganda
Source: Int J Environ Res Public Health. 2020 Aug 24;17(17):6129. doi: 10.3390/ijerph17176129 (PMC7504603; doi:10.3390/ijerph17176129)
Supplement: Supplementary file 1 [file ijerph-17-06129-s001.pdf]

International Journal of
Environmental Research and Public Health

MDPI

Table S1. Comparison of baseline characteristics between 1440 participants with complete records and 458 with missing records.

Variable	Categories	Frequency (N = 1440) n (col %)	Missing N = 458 n (col %)	Chi-Square/ Fisher's Exact P-value
Age (Years)				
	15C19	360 (25.0)	159 (34.7)	<0.001
	20-24	1080 (75.0)	299 (65.3)	
Education level				
	None	82 (5.7)	17 (3.7)	0.04
	Less than secondary	1115 (77.4)	344 (75.1)	
	Secondary or higher	243(16.9)	97 (21.2)	
Marital status				
	Single (never married)	746 (51.8)	266 (58.1)	0.011
	Married	61 (4.2)	26 (5.7)	
	Widowed/separated	633 (44)	166 (36.2)	
Number of biological children [a]				
	None	435(30.2)	-	
	One	586 (40.7)	-	
	>1	419 (29.1)	-	
Ever used illicit drugs [b]				
	Yes	505 (35.1)	-	
	No	935 (64.9)	-	
Ever tested for HIV [c]				
	Yes	1325 (92.0)	-	
	No	115 (8.0)	-	
Last HIV test [d]				
	≤ 6 months	954 (72.0)	-	
	> 6 months	371 (28.0)	-	
HIV status				
	Positive	297 (20.6)	88 (19.2)	0.513
	Negative	1143 (79.4)	370 (80.8)	
Main job [e]				
	Sex work	972 (67.5)	-	
	Other job	468 (32.5)	-	
Paying sexual partners in past one month [f]				
	< 10 partners	291 (20.2)	-	
	≥ 10 partners	1149 (79.8)	-	
Condom use with paying partners in past month [g]				
	Consistent	707 (49.1)	-	
	Inconsistent	733 (50.9)	-	
Ever experienced IPV [h]				
	Yes	662 (46)	-	
	No	778 (54)	-	
Contraceptive use [i]				
	Yes	964 (66.9)	-	
	No	476 (33.1)	-	

[a, b, c, d, e, f, g, h, i] 458 participants had missing data on ≥ one variables and were excluded from the analysis

Table S2. Imputed models for factors associated with AUD (N = 1898).

		AUDIT ≥8		AUDIT ≥16	
**Variable**	**Sub Category**	**Adjusted OR (95%CI)**	**p value**	**Adjusted OR (95%CI)**	**p value**
Age (years)					
	15–17	1.00	**<0.001**	1.00	**0.006**
	20–24	**1.60 (1.29–2.00)**		**1.44 (1.11–1.87)**	
Education level					
	Less than secondary	1.00	0.686	1.00	0.874
	Secondary or higher	1.05 (0.82–1.35)		0.98 (0.73–1.30)	
Marital Status					
	Single never married	-		1.00	
	Married	-		0.99 (0.58–1.70)	0.983
	Separated or widowed	-		1.04 (0.82–1.30)	0.755
Biological children					
	None	-		-	
	≥1	-		-	
Current illicit drug use					
	No	1.00	**<0.001**	1.00	**<0.001**
	Yes	**3.37 (2.66–4.29)**		**2.99 (2.39-3.76)**	
Ever experienced IPV					
	No	1.00	**0.001**	1.00	**<0.001**
	Yes	**1.38 (1.13–1.68)**		**1.85 (1.49-2.31)**	
Paying partners (past month)					
	<10	1.00	**0.031**	1.00	0.086
	≥10	1.29 (1.02–1.63)		1.28 (0.97–C1.70)	
Condom use with paying sexual partners					
	Consistent	1.00	**0.006**	1.00	**0.001**
	Inconsistent	**1.32 (1.08-1.61)**		**1.43 (1.15–C1.78)**	
Contraceptive use					
	No	1.00	0.537	**-**	
	Yes	0.93 (0.75–C1.16)		**-**	
HIV status					
	Positive	1.00	**0.01**	1.00	0.874
	Negative	**1.38 (1.08–C1.77)**		1.09 (0.84–C1.43)	
